# Supplementary material for: Modeling of Charge Injection, Recombination, and Diffusion in Complete Perovskite Solar Cells on Short Time Scales
Source: Materials (Basel). 2023 Nov 10;16(22):7110. doi: 10.3390/ma16227110 (PMC10672245; doi:10.3390/ma16227110)
Supplement: Supplementary file 1 [file materials-16-07110-s001.zip › materials-2693068-supplementary.pdf]

Supporting Information

Modeling of Charge Injection, Recombination, and Diffusion in Complete Perovskite Solar Cells on Short Time Scales

Krzysztof Szulc [A], *Katarzyna Pydzińska-Białek* [B] *and Marcin Ziółek* [B]*

[A] Institute of Spintronics and Quantum Information, Faculty of Physics, Adam Mickiewicz University, Poznań, Uniwersytetu Poznańskiego 2, 61-614 Poznań, Poland

[B] Institute of Physics, Faculty of Physics, Adam Mickiewicz University, Poznań, Uniwersytetu Poznańskiego 2, 61-614 Poznań, Poland

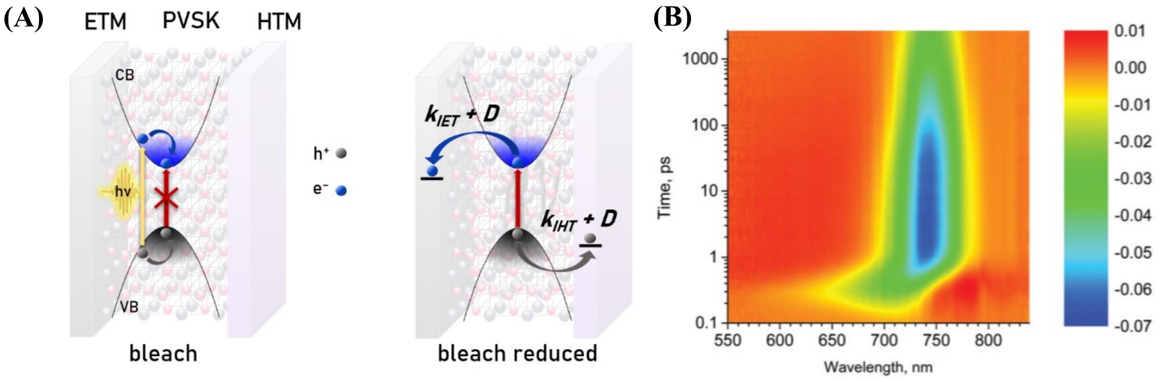

Figure S1. (A) Schematic presentation of the formation of the bleach (blocked transition) at the low-energy edge of absorption in perovskite after charge cooling (left), and the reduction of bleach after either electron transfer to ETM or hole transfer to HTM due to the delocalization of the charges (right). (B) Pseudo-color 2D spectra of original transient absorption data corresponding to higher pump fluence excited from ETM side in Figure 2C. The negative bleach signal is indicated by yellow, green, and blue.

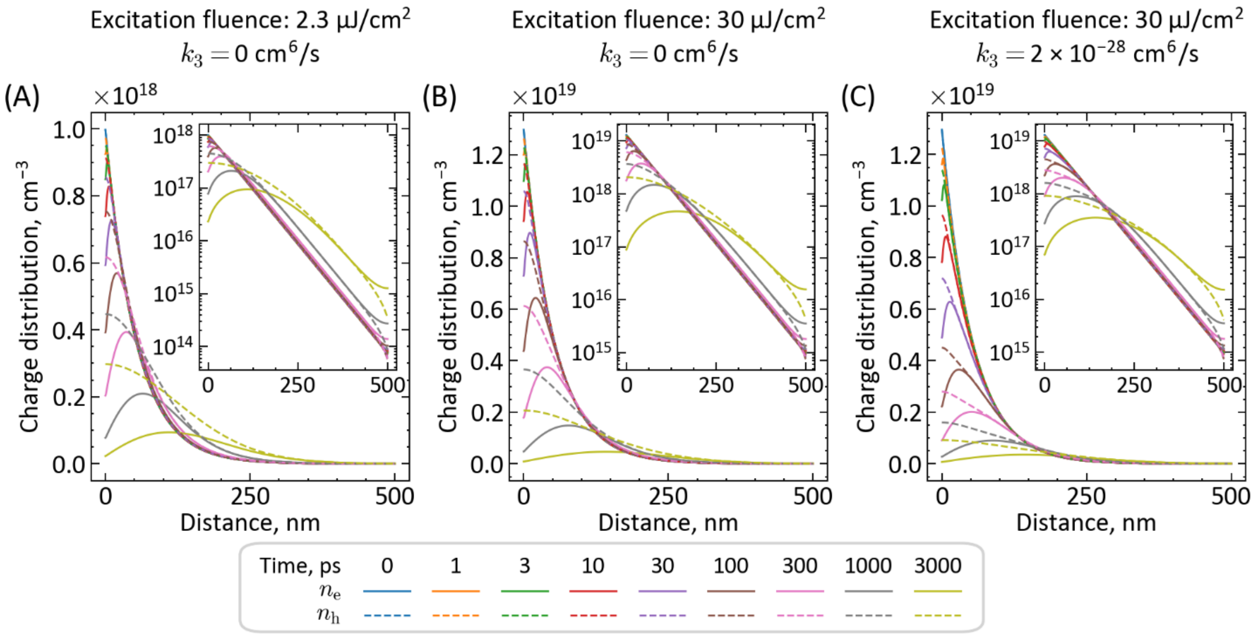

Figure S2. Simulation of the distribution of the electrons and holes along perovskite thickness at different times after excitation from the ETM side for low and high pump fluence. If not otherwise indicated in the graph insets, the values of the parameters for the simulations are the following: L=500 nm, χ=0.7, D=2×10^{-2} cm^2/s, k_1=1×10^5 s^{-1}, k_2=2×10^{-10} cm^3/s, k_{IET}=k_{IHT}=1×10^{11} s^{-1}, α=190 000 cm^{-1} (475 nm excitation from ETM side). The left column shows the results for pump fluence of 2.3 μJ/cm^2 and k_3=0, the middle column for 30 μJ/cm^2 and k_3=0, and the right column for 30 μJ/cm^2 and k_3=2×10^{-28} cm^6/s.

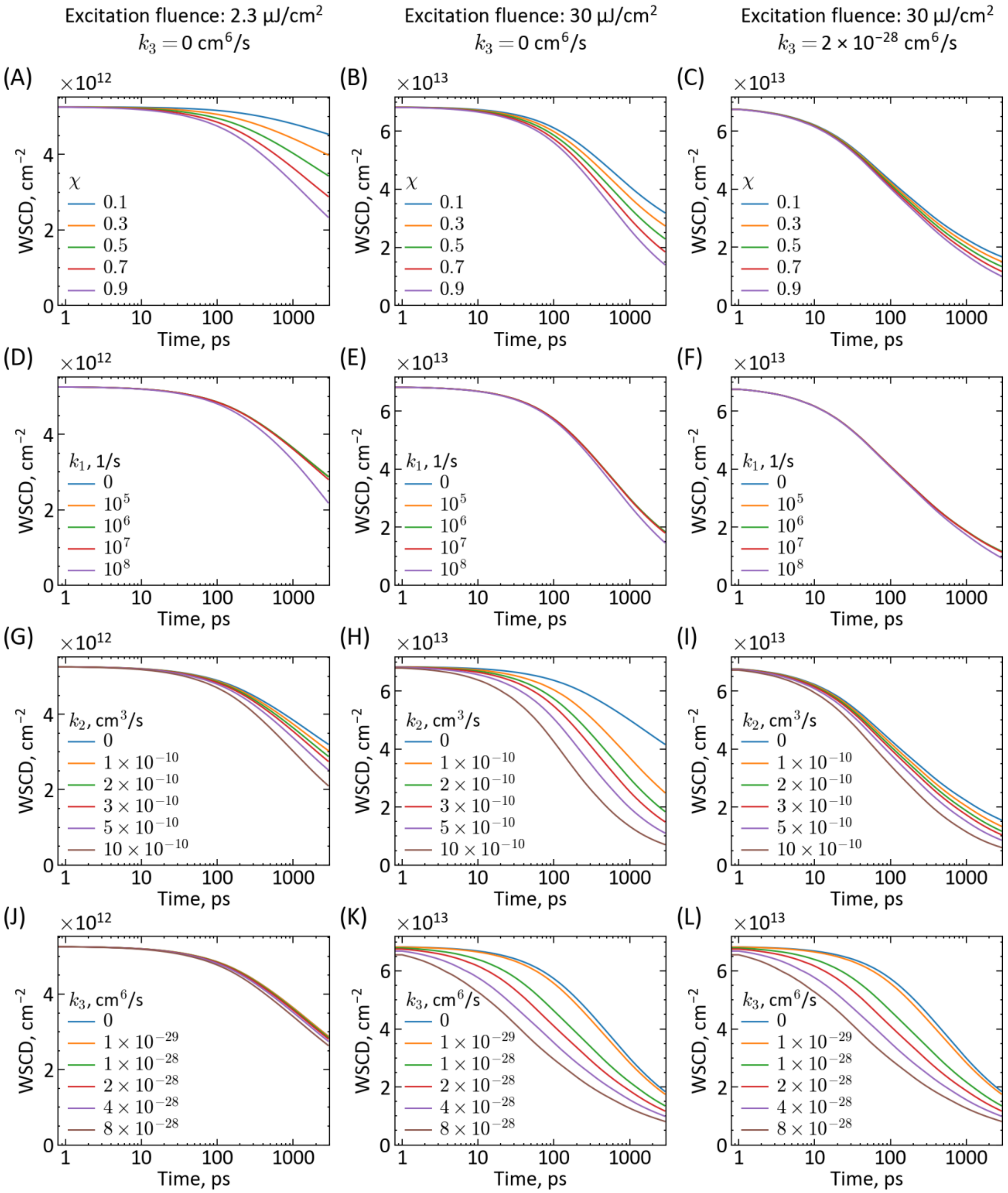

Figure S3. Simulations of charge population decay observed in transient absorption of PSCs (calculated from eq. (10)) for different parameters variation (χ, k_1, k_2, k_3). If not otherwise indicated in the graph insets, the values of the parameters for the simulations are the following: L=500 nm, χ=0.7, D=2×10^{-2} cm^2/s, k_1=1×10^5 s^{-1}, k_2=2×10^{-10} cm^3/s, k_{IET}=k_{IHT}=1×10^{11} s^{-1}, α=190 000 cm^{-1} (475 nm excitation from ETM side). The left column shows the results for pump fluence of 2.3 μJ/cm^2 and k_3=0, the middle column for 30 μJ/cm^2 and k_3=0, and the right column for 30 μJ/cm^2 and k_3=2×10^{-28} cm^6/s.

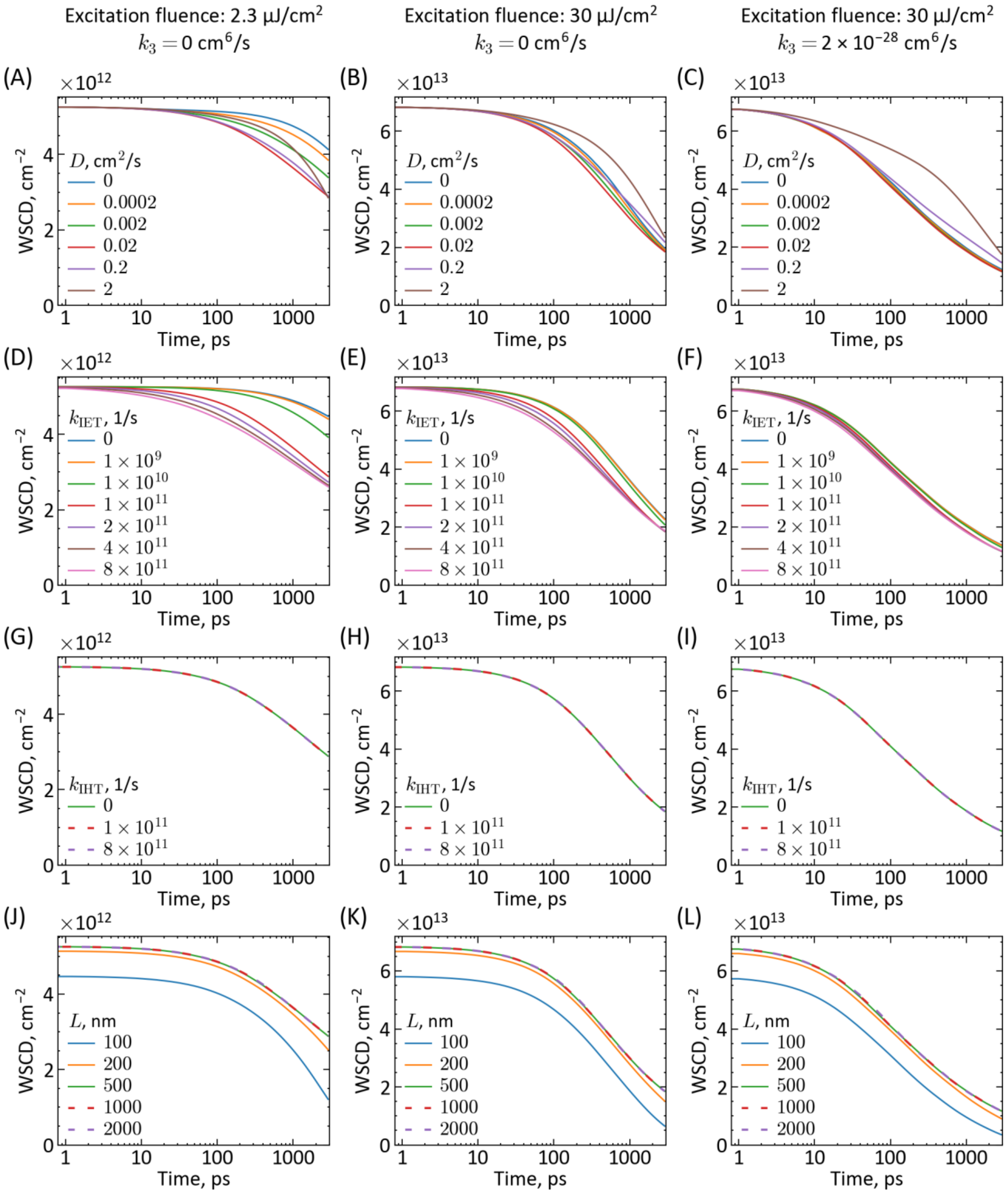

Figure S4. Simulations of charge population decay observed in transient absorption of PSCs (calculated from eq. (10)) for different parameters variation (D, k_{IET}, k_{IHT}, L). If not otherwise indicated in the graph insets, the values of the parameters for the simulations are the following: L=500 nm, χ=0.7, D=2×10^{-2} cm^2/s, k_1=1×10^5 s^{-1}, k_2=2×10^{-10} cm^3/s, k_{IET}=k_{IHT}=1×10^{11} s^{-1}, α=190 000 cm^{-1} (475 nm excitation from ETM side). The left column shows the results for pump fluence of 2.3 μJ/cm^2 and k_3=0, the middle column for 30 μJ/cm^2 and k_3=0, and the right column for 30 μJ/cm^2 and k_3=2×10^{-28} cm^6/s.

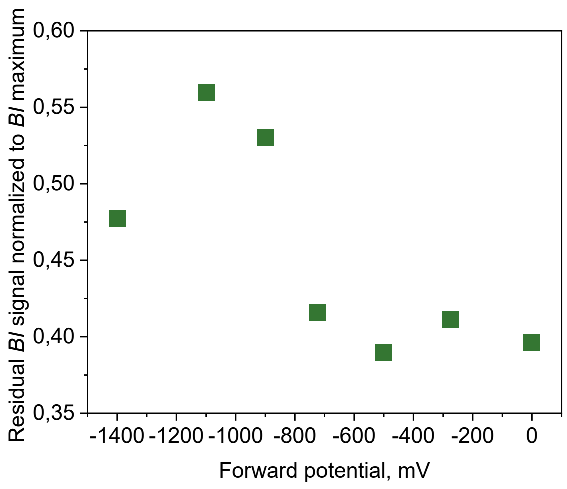

Figure S5. Averaged residual *BI* signal for a different applied voltage of PSC excited from ETM side with 475 nm pump pulse of 2.3 μJ/cm². The change reflects the variation in electron injection rate constant (k_{IET}) for different potential values as all other parameters in our model are the same. Due to several measurements in the same spot, some photoinduced changes probably occurred that slightly change the kinetics, so we minimize this effect by averaging two measurements for the same voltage: one for voltage increasing and one for voltage decreasing scan. Then the data were averaged for two such series. That's why the residual values are slightly smaller than that in Figure 3B measured over a much shorter time.

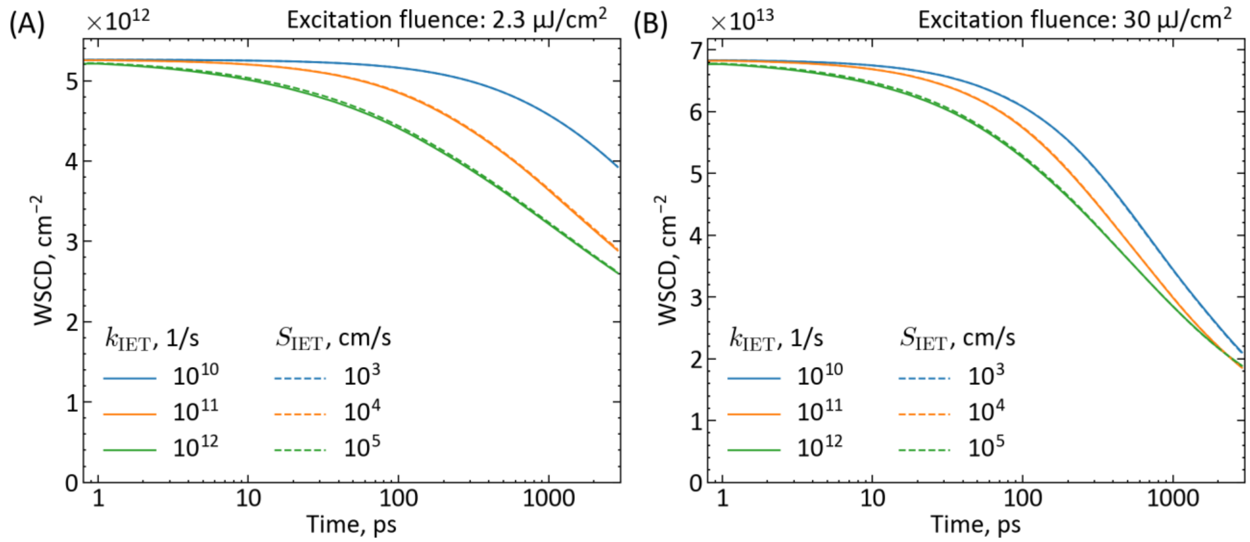

Figure S6. The comparison between two approaches of interface charge transfer implementation—using delta functions in the vicinity of the boundary (solid lines) and using boundary conditions for the gradient of the charge population (dashed lines) for three values of k_{IET} and S_{IET}. The values of the parameters for the simulations are the following: L=500 nm, χ=0.7, D=2×10^{-2} cm²/s, k_1=1×10^5 s^{-1}, k_2=2×10^{-10} cm³/s, k_3=0, k_{IHT}=1×10^{11} s^{-1}, α =190 000 cm^{-1} (475 nm excitation from ETM side). The left plot (A) shows the results for pump fluence of 2.3 μJ/cm² and the right plot (B) for 30 μJ/cm².
